# Partial Replacement of Corn with Jackfruit Silage Maintains Productive Performance and Carcass Characteristics of Feedlot Lambs

**DOI:** 10.3390/ani16172667

**Published:** 2026-08-25

**Authors:** Michelle Patricia Frazer Salt, Leandro Silva Nascimento, Hérick Pachêco Rodrigues, Joyanne Mirelle Sousa Ferreira, Lígia Lins Souza, Cristiane Leal dos Santos Cruz, Robério Rodrigues Silva, Gleidson Giordano Pinto de Carvalho, Stefanie Alvarenga Santos, Ícaro dos Santos Cabral, Flávio Moreira de Almeida, José Augusto Gomes Azevêdo

**Affiliations:** 1 Postgraduate Program in Animal Science, Universidade Estadual do Sudoeste da Bahia (UESB), Itapetinga 45700-000, Bahia, Brazil; michellefrazersalt@yahoo.com.br (M.P.F.S.); sousajoyanne@gmail.com (J.M.S.F.); 2 Department of Educational Support, Instituto Federal de Educação, Ciência e Tecnologia de Rondônia (IFRO), Ariquemes 76870-000, Rondônia, Brazil; lsn_19@hotmail.com.br; 3 Postgraduate Program in Animal Science, Universidade Estadual de Santa Cruz (UESC), Ilhéus 45662-900, Bahia, Brazil; hprodrigues.ppgca@uesc.br (H.P.R.); icaro.cabral@agro.gov.br (Í.d.S.C.); flavioma@unipam.edu.br (F.M.d.A.); 4 Department of Exact and Technological Sciences, Universidade Estadual do Sudoeste da Bahia (UESB), Vitória da Conquista 45083-900, Bahia, Brazil; ligia.souza@uesb.edu.br; 5 Department of Rural and Animal Technology, Universidade Estadual do Sudoeste da Bahia (UESB), Itapetinga 45700-000, Bahia, Brazil; cleal@uesb.edu.br (C.L.d.S.C.); roberio@uesb.edu.br (R.R.S.); 6 School of Veterinary Medicine and Animal Science, Universidade Federal Bahia (UFBA), Salvador 40170-110, Bahia, Brazil; gleidsongiordano@ufba.br (G.G.P.d.C.); stefanie.alvarenga@ufba.br (S.A.S.); 7 Ministério da Agricultura e Pecuária (MAPA), JBS S/A, Rodovia BA-263, km 167, Zona Rural, Itapetinga 45700-000, Bahia, Brazil; 8 Veterinary Medicine Program, Centro Universitário de Patos de Minas (UNIPAM), Patos de Minas 38700-207, Minas Gerais, Brazil; 9 Department of Agricultural and Environmental Sciences, Universidade Estadual de Santa Cruz (UESC), Ilhéus 45662-900, Bahia, Brazil

**Keywords:** *Artocarpus heterophyllus*, alternative feedstuff, feed-food competition, ruminal fermentation, small ruminants

## Abstract

Dependence on corn may increase feed costs and supply vulnerability in tropical livestock systems. This study evaluated replacing corn with whole-fruit jackfruit (*Artocarpus heterophyllus* Lam.) silage in feedlot lamb diets. Sixteen castrated male Santa Inês crossbred lambs (26.2 ± 3.6 kg) received diets in which jackfruit silage replaced 0, 333, 666, or 1000 g/kg of corn dry matter in the concentrate. Jackfruit silage had lower non-fibrous carbohydrate gas volume (132.9 vs. 182.9 mL), degradation rate (0.081 vs. 0.096 h^−1^), and total carbohydrate gas volume (238.5 vs. 298.2 mL) than corn (*p* < 0.05). Dry matter intake increased linearly, whereas organic matter digestibility, final body weight, and total weight gain decreased linearly as replacement increased (*p* ≤ 0.011). Feed conversion ratio increased from 6.6 to 12.3 kg dry matter intake/kg gain. Nevertheless, 333 g/kg replacement maintained 98.5% of final body weight and 96.0% of total weight gain. Carcass yields and Longissimus muscle area were unaffected. One-third replacement reduced corn use by 26.2% per kilogram of gain while maintaining productive viability. Jackfruit silage is therefore a strategic partial, rather than complete, substitute for corn in tropical sheep diets.

## Simple Summary

Corn is widely used in lamb diets, but its price and availability can limit livestock production in tropical regions. Jackfruit is abundant in many tropical areas, and whole fruits that are not used for human consumption can be preserved as silage and used as animal feed. This study tested whether jackfruit silage could replace corn in the concentrate offered to feedlot lambs. Complete replacement reduced growth and worsened feed conversion. However, replacing one-third of the corn maintained 98.5% of final body weight and 96.0% of total weight gain compared with the conventional corn-based diet. Carcass yield and the area of the main loin muscle were also maintained. At this replacement level, the amount of corn dry matter required per kilogram of body weight gain was reduced by 26.2%. These results show that jackfruit silage should not be considered a complete substitute for corn, but it can be used strategically at a moderate level. This approach can transform an underused tropical biomass into feed, reduce dependence on conventional grain, and support more diversified feeding systems for sheep in regions where corn is costly or scarce.

## 1. Introduction

Livestock production systems in tropical and subtropical regions increasingly rely on conventional feed grains, particularly corn, as major sources of dietary energy [1,2]. Although corn supports high levels of animal productivity, dependence on a single feed commodity may expose livestock systems to feed shortages, market instability, and supply chain disruptions. This vulnerability may be intensified by geopolitical instability, climate variability, and increasing competition among food, feed, and industrial uses [1,2]. Concentrated international feed supply chains can further increase the exposure of livestock systems to fluctuations in grain availability and prices [2,3]. These challenges have intensified interest in locally available feed resources that can reduce dependence on conventional grains while maintaining animal productivity [4,5].

In this context, the utilization of locally available biomass resources has emerged as an important strategy for improving resource-use efficiency and strengthening the resilience of livestock production systems. Agricultural residues, agro-industrial by-products, and underutilized tropical biomasses can reduce dependence on conventional feed grains, decrease feed-food competition, and contribute to circular bioeconomy approaches in animal production [4,5,6]. Furthermore, feed diversification has been increasingly recognized as a key component of resilient livestock systems because it reduces vulnerability to fluctuations in feed availability and market prices while maintaining productive viability [1,7]. Accordingly, identifying biologically viable substitution levels, rather than maximizing the replacement of conventional ingredients, may represent a practical strategy to balance productive performance and reduced reliance on external feed resources.

Among the underutilized tropical biomasses with potential for animal feeding, jackfruit (*Artocarpus heterophyllus* Lam.) has attracted increasing attention because of its high production potential and broad adaptation to tropical environments. Under favorable conditions, individual trees may produce up to 700 fruits annually, resulting in yields ranging from 50 to 80 t/ha [8]. Despite this substantial production, a large proportion of the fruit remains underutilized, as non-edible fractions may represent approximately 50–75% of total fruit mass depending on processing conditions [9,10,11]. Consequently, considerable quantities of residues are generated during harvesting and processing, with estimates indicating that nearly 2.96 million tons of jackfruit-derived material may be available annually from peels and seeds alone [12]. Recent reviews have highlighted the potential of these residues as livestock feed resources capable of supporting animal production, reducing feed costs, and promoting the valorization of biomass that would otherwise remain underexploited [8,13].

The chemical composition of jackfruit-derived feeds varies according to the material evaluated [14,15,16]. Pereira et al. [14] reported NDF concentrations of 271–272 g/kg DM and CP concentrations of 63–68 g/kg DM in mature flint and soft whole fruits, whereas Suma et al. [15] reported NDF concentrations of 341–361 g/kg DM and CP concentrations of 88–95 g/kg DM in jackfruit residue and unripe whole-fruit silages. Azevêdo et al. [16] further reported marked compositional differences between jackfruit and jackfruit silage, with corrected NDF concentrations of 427 and 161 g/kg DM and NFC concentrations of 356 and 698 g/kg DM, respectively. These findings demonstrate that jackfruit-derived feeds should not be characterized by a single fixed fiber, protein, or carbohydrate profile. Rather, their nutritional value should be interpreted according to the specific material evaluated, supporting their consideration as alternative feed resources [15] and, in some cases, as partial substitutes for conventional energy concentrates [16]. Furthermore, the soluble carbohydrates present in jackfruit have been associated with improved fermentation during ensiling, including enhanced lactic fermentation, reduced fermentative losses, greater dry matter recovery, and increased degradability of fibrous fractions, thereby facilitating biomass preservation and year-round utilization of this seasonal tropical resource [17,18].

Despite the nutritional potential demonstrated for different jackfruit-derived materials, the available evidence remains distributed across distinct feed materials and experimental objectives. Studies conducted by Thi Mui et al. [19], Das and Ghosh [20], and Thanh et al. [21] evaluated jackfruit foliage in goat diets, primarily as a forage resource, and therefore provide limited information regarding the nutritional value of whole-fruit jackfruit silage. In contrast, studies by Pereira et al. [14], Suma et al. [15], Azevêdo et al. [16], Santos et al. [17], and Dórea et al. [18] characterized the chemical composition, fermentation profile, or nutritive attributes of jackfruit-derived materials but did not evaluate the combined effects of whole-fruit jackfruit silage on nutrient utilization, productive performance, and carcass responses under practical feeding conditions. Consequently, it remains unclear to what extent whole-fruit jackfruit silage can partially replace corn in feedlot lamb diets without compromising productive performance and carcass value.

We hypothesized that partial replacement of corn with jackfruit silage would maintain productive performance and carcass characteristics while reducing dependence on a conventional feed grain. Therefore, this study aimed to evaluate the effects of replacing corn with jackfruit silage on in vitro ruminal fermentation kinetics, nutrient intake, apparent digestibility, growth performance, carcass characteristics, commercial cuts, and non-carcass components of feedlot lambs.

## 2. Materials and Methods

### 2.1. Ethical Approval and Experimental Location

The experimental procedures, including the use of the two rumen-cannulated donor sheep for ruminal inoculum collection, were conducted in accordance with the ethical principles for animal experimentation adopted by the Ethics Committee on Animal Use of the Universidade Estadual de Santa Cruz (UESC), Ilhéus, Bahia, Brazil, and were approved under protocol no. 012/11 on 13 September 2011.

The in vitro assay and the animal experiment were conducted at the Ruminant Nutrition and Feeding Research Laboratory, UESC, Ilhéus, Bahia, Brazil (14°47′ S, 39°17′ W; 57 m above sea level). According to the Köppen climate classification system, the region is classified as Af (humid tropical), with an average annual temperature of approximately 24.5 °C and an average annual precipitation of approximately 1700 mm.

### 2.2. In Vitro Ruminal Fermentation

An in vitro ruminal fermentation assay was conducted using the semi-automated gas production technique described by Maurício et al. [22]. The jackfruit silage sample used as substrate in the in vitro assay was obtained from the same ensiled material used to prepare the experimental diets in the animal trial. Samples of jackfruit silage and ground corn were dried and ground to pass through a 1 mm screen. A 300 mg sample of each substrate was weighed into each 50 mL glass fermentation flask. Four fermentation flasks were prepared per substrate, and each flask was considered an experimental unit for the in vitro comparison. Prior to incubation, all flasks were autoclaved, dried, and flushed with CO_2_ to establish anaerobic conditions.

The culture medium was prepared as described by Theodorou et al. [23]. Each flask received 28.125 mL of buffered mineral solution and 3.125 mL of ruminal inoculum, resulting in a final incubation volume of 31.25 mL. The flasks were sealed with butyl rubber stoppers and incubated at 39 °C.

The buffered mineral solution, including macro- and micromineral components, was prepared according to Menke and Steingass [24]. The solution was prepared approximately 2 h before incubation and maintained at 39 °C under continuous CO_2_ flushing and constant stirring to ensure homogeneity. The pH was continuously monitored and adjusted to 6.8 before inoculation.

Ruminal inoculum was obtained from two rumen-cannulated Santa Inês crossbred sheep (approximately 50 kg body weight) maintained under identical management conditions and fed elephant grass silage together with the concentrate formulation corresponding to the control diet (0 g/kg jackfruit silage replacement). Ruminal contents were collected approximately 4 h after morning feeding, corresponding to the post-prandial period of active fermentation. Equal proportions of ruminal fluid from the two donor animals were combined to form a single pooled inoculum. The pooled inoculum was homogenized and used uniformly across all fermentation flasks within the incubation run. The same pooled inoculum was used for all substrates to ensure that corn and jackfruit silage were compared under identical microbial and incubation conditions. The pooled ruminal fluid was filtered through four layers of cheesecloth under continuous CO_2_ flushing, transferred to pre-warmed thermal containers maintained at 39 °C, and immediately transported to the laboratory. The inoculum was maintained under anaerobic conditions until use in the experiment.

After inoculation, the flasks were sealed with butyl rubber stoppers, gently shaken, and incubated at 39 °C. Blank flasks containing only buffered inoculum and culture medium were included to correct for gas production originating from the inoculum. Tifton 85 hay was used as a reference substrate to verify the consistency of fermentation.

Gas pressure generated in the headspace of the flasks was measured using a digital pressure transducer and data acquisition system (Pressure Press Data 800, LANA/CENA-USP, Piracicaba, SP, Brazil) coupled to a 0.6 mm needle and a 6 mL syringe. Measurements were obtained at 1, 2, 3, 4, 6, 8, 10, 12, 14, 17, 20, 24, 28, 36, 48, 60, and 72 h after incubation.

Pressure values (P, psi) were converted into gas volume (mL) using the regression equation proposed by Santos et al. [25], following the methodology described by Maurício et al. [22]:

V (mL) = 0.04755 + 1.9754P + 0.01407P^2^ (R^2^ = 0.99)

where V is the gas volume (mL) and P is the pressure measured within the fermentation flask.

Cumulative gas production data obtained throughout the 72 h incubation period were used to fit the bicompartmental logistic model proposed by Schofield et al. [26] as follows:

$$V\left(t\right)=\frac{FCGV}{1+\exp\left[2-4\times FCDR\times \left(t-L\right)\right]}+\frac{NFCGV}{1+\exp\left[2-4\times NFCDR\times \left(t-L\right)\right]}$$

where V(t) is the cumulative gas volume (mL) at incubation time t (h); FCGV is the asymptotic gas volume derived from the fibrous carbohydrate (FC) fraction (mL); FCDR is the fractional gas production rate of the FC fraction (h^−1^); NFCGV is the asymptotic gas volume derived from the non-fibrous carbohydrate (NFC) fraction (mL); NFCDR is the fractional gas production rate of the NFC fraction (h^−1^); and L is the common lag time for gas production (h).

Total asymptotic gas volume (mL) was calculated as the sum of FCGV and NFCGV.

### 2.3. Animals and Experimental Design

The lambs were purchased from Fazenda Santa Cruz da Vitória, a commercial farm located in the municipality of Santa Cruz da Vitória, Bahia, Brazil. Sixteen castrated male Santa Inês crossbred lambs, approximately four months of age, with an initial body weight of 26.2 ± 3.6 kg (mean ± standard deviation), were used. The animals were housed individually in suspended pens (0.80 × 1.20 m) equipped with slatted wooden floors, individual feeders, and automatic drinkers, with free access to water throughout the study. All 16 lambs completed the experimental period, and no animals were lost or excluded from the statistical analyses.

Before the beginning of the trial, all lambs were identified, vaccinated against clostridial diseases using Sintoxan Polivalente F (Boehringer Ingelheim Animal Health, Campinas, SP, Brazil), treated for internal and external parasites according to the manufacturer’s recommendations, and supplemented with vitamins A, D, and E.

The experiment was conducted using a completely randomized design with four dietary treatments and four animals per treatment. Each lamb was considered an experimental unit and randomly assigned to one of the experimental diets.

The study lasted 85 d, comprising a 15 d adaptation period to the facilities and experimental diets and a subsequent 70 d data-collection period. Feed was offered twice daily at 08:00 and 16:00 h. Orts were maintained at approximately 10% of the feed offered to ensure ad libitum intake throughout the experiment.

### 2.4. Experimental Diets and Feed Management

The experimental diets were formulated to be isonitrogenous, containing approximately 156 g crude protein (CP)/kg DM and a forage-to-concentrate ratio of 40:60 on a DM basis (Table 1). Diets were formulated according to NRC [27] recommendations for growing lambs with an initial body weight of approximately 26 kg and a target average daily gain of 200 g/d.

Elephant grass silage (*Pennisetum purpureum* Schum.) was used as the forage source and was prepared with the addition of 100 g/kg corn bran on a fresh matter basis. The concentrate consisted of ground corn, soybean meal, urea, and a commercial mineral supplement for sheep. Ground corn was progressively replaced with jackfruit silage at four inclusion levels.

Corn was replaced by jackfruit silage at 0, 333, 666, and 1000 g/kg of corn DM, corresponding to 0, 33.3, 66.6, and 100% replacement. These levels were selected to provide an approximately equally spaced progression from no replacement to complete replacement of corn, corresponding to 0, approximately one-third, approximately two-thirds, and complete replacement. This treatment structure allowed the biological response across the full replacement range to be evaluated and supported the assessment of linear and quadratic response patterns using orthogonal polynomial contrasts.

Jackfruit (*Artocarpus heterophyllus* Lam.) was harvested from a rural property located in the municipality of Ubatã, Bahia, Brazil, and ensiled as whole fruit, including the peel, pulp, and seeds. Fruits were selected at an intermediate pre-ripening stage, being neither fully green nor fully ripe. The whole fruits were chopped into approximately 2 cm pieces and progressively packed into 28 plastic drums with a nominal capacity of 120 L. Approximately 78 kg of fresh chopped jackfruit were packed into each drum, corresponding to an estimated fresh-matter packing density of approximately 650 kg/m^3^. During filling, the material was added gradually in successive layers and compacted by trampling to reduce trapped air. No microbial inoculant or other ensiling additive was used. After filling, the drums were closed with screw-top lids and remained sealed for 56 d of fermentation before the beginning of use. Thereafter, the drums were opened sequentially according to the daily requirements of the feeding trial. Across the experimental sampling periods, the mean chemical composition of the jackfruit silage was 210 g/kg fresh matter DM and, on a DM basis, 939 g/kg OM, 80 g/kg CP, 46 g/kg EE, 424 g/kg NDF, and 389 g/kg NFC. A sample of the same ensiled material used to prepare the experimental diets was used as the jackfruit silage substrate in the in vitro assay.

The experimental diets were mixed daily immediately before feeding. Orts were collected and weighed each morning before feed delivery. Samples of feeds offered and refusals were collected every 14 d, pooled by animal within period, stored at −20 °C, and subsequently analyzed for chemical composition. Dietary DM concentration was monitored weekly and used to adjust the feed allowance throughout the experimental period. Because jackfruit silage had a lower DM concentration than ground corn, the fresh-matter moisture content of the diets increased progressively with jackfruit silage inclusion. Feed allowance and intake calculations were therefore adjusted and expressed on a DM basis.

### 2.5. Dry Matter and Organic Matter Intake and Apparent Organic Matter Digestibility

Feed refusals were collected and weighed daily before morning feeding. Representative samples of the feeds offered and refusals were collected daily, composited every 14 d, identified, and stored at −20 °C until analysis.

Dry matter intake (DMI) and organic matter intake (OMI) were calculated as the difference between feed offered and refusals on a dry matter basis. All feed intake values reported in the manuscript were calculated and expressed on a DM basis.

Fecal samples were collected directly from the rectum over five consecutive days during three sampling periods (days 20–24, 45–49, and 65–69 of the experimental phase). Samples were oven-dried at 60 ± 5 °C for 72 h, ground through a 1 mm screen Wiley-type mill (TE-650, Tecnal, Piracicaba, SP, Brazil), and stored for subsequent analyses.

Fecal dry matter output was estimated using indigestible acid detergent fiber (iADF) as an internal marker according to Casali et al. [29]. Feed, refusals, and fecal samples were placed in nonwoven textile bags (100 g/m^2^) at a ratio of 20 mg DM/cm^2^ and incubated in the rumen of a cannulated bovine for 288 h. After incubation, bags were washed thoroughly under running water, oven-dried at 60 ± 5 °C for 72 h and processed according to Detmann et al. [30] for determination of iADF concentration.

Fecal dry matter output was calculated as:

FDMO (g/d) = marker intake (g/d)/marker concentration in feces (g/g)

Apparent organic matter digestibility was calculated as:

Organic matter digestibility (g/kg) = [(organic matter intake − fecal organic matter output)/organic matter intake] × 1000.

### 2.6. Chemical Analyses

Samples of feeds, refusals, and feces were dried at 60 ± 5 °C for 72 h and ground through a 1 mm screen Wiley-type mill (TE-650, Tecnal, Piracicaba, SP, Brazil). Dry matter (DM), crude protein (CP), ether extract (EE), and ash contents were determined according to the Association of Official Analytical Chemists [31].

Neutral detergent fiber was determined according to Van Soest et al. [32], using heat-stable α-amylase. The values were corrected for residual ash and protein and were expressed as NDFap, following Mertens et al. [33]. Acid detergent fiber was determined according to Van Soest et al. [32]. Neutral detergent insoluble nitrogen was determined according to Licitra et al. [34] and used for protein correction of the NDF fraction. Non-fibrous carbohydrates (NFC) were calculated according to Hall [35], as follows:

NFC = 100 − MM − EE − NDFap − (CP − CPu + U)

where MM is mineral matter, EE is ether extract, NDFap is neutral detergent fiber corrected for ash and protein, CPu is the crude protein equivalent supplied by urea, and U is the dietary urea concentration, with all components expressed as percentage of DM. The total digestible nutrients (TDN) were estimated according to Cruz et al. [28].

### 2.7. Performance Measurements, Slaughter Procedures and Carcass Traits

Lambs were weighed individually at the beginning and end of the experimental period after a 16 h fast from solid feed, with free access to water. Initial body weight (IBW) and final body weight (FBW) were recorded using a scale designed for small ruminants (BL300Pro, Laboremus, Campina Grande, PB, Brazil) with a precision of 200 g. The feed offered and refusals were weighed daily throughout the experiment. Dry matter intake (DMI) was calculated as the difference between feed offered and refusals and expressed as g/day. Total weight gain (TWG) was calculated as the difference between final and initial body weights. Feed conversion ratio (FCR) was calculated as total dry matter intake over the experimental period divided by total body weight gain and expressed as kg DM intake/kg gain.

At the end of the experimental period, lambs were fasted from solid feed for 16 h with free access to water and subsequently weighed to determine slaughter body weight (SBW). Animals were stunned and slaughtered by exsanguination in accordance with the procedures approved by the Institutional Animal Ethics Committee. After evisceration, the gastrointestinal tract was weighed full and empty, and empty body weight (EBW) was calculated as follows:

EBW = SBW − gastrointestinal content

Hot carcass weight (HCW) was recorded immediately after slaughter, and hot carcass yield (HCY) was calculated as HCW/SBW × 100.

The carcasses were chilled at 4 ± 1 °C for 24 h, after which the cold carcass weight (CCW) was recorded. The cold carcass yield (CCY) was calculated as CCW/SBW × 100, and the chilling loss (CL) was calculated as:

CL = [(HCW − CCW)/HCW] × 100

After chilling, carcasses were longitudinally split, and the left half-carcass was used for morphometric evaluation. Internal carcass length, external carcass length, leg length, leg width, leg depth, chest depth, rump width, rump perimeter, and left half-carcass weight were recorded.

The *Longissimus lumborum* muscle exposed between the 12th and 13th ribs was used for carcass measurements. The rib eye area was determined using graph paper, and the subcutaneous fat thickness was measured over the *Longissimus lumborum* muscle at the same anatomical location using a digital caliper (Eccofer, João Pessoa, PB, Brazil) with a resolution of 0.01 mm.

Muscle pH and carcass internal temperature were measured in the *Longissimus lumborum* muscle at the 12th rib interface approximately 30 min postmortem and after 24 h of chilling. Muscle pH and carcass internal temperature were measured using a calibrated portable pH/temperature meter equipped with a combined penetration pH electrode and temperature probe (Testo 205, Testo SE & Co. KGaA, Lenzkirch, Germany). The probe was inserted approximately 2.5 cm into the Longissimus lumborum muscle.

### 2.8. Non-Carcass Component Analysis

Non-carcass components were evaluated immediately after slaughter and after evisceration. The components assessed included blood, skin, head, hooves, tail, heart, kidneys, spleen, lungs, tongue, internal fat, and the empty gastrointestinal tract (GIT). All components were individually weighed using a digital scale with a precision of ±5 g.

The head, hooves, tail, and visceral organs were removed according to the anatomical procedures described by Cezar and Sousa [36]. Internal fat, consisting of mesenteric and perirenal adipose tissues, was separated and weighed individually. The gastrointestinal tract was emptied, washed, and weighed to obtain the empty GIT weight. All non-carcass components were expressed as absolute weight (kg).

### 2.9. Statistical Analysis

Data were analyzed using SAS version 9.4 (SAS Institute Inc., Cary, NC, USA) [37]. Residuals were evaluated for normality using the Shapiro–Wilk test and for homogeneity of variance using Levene’s test. All variables met the assumptions of normality and homoscedasticity, and no data transformation was required.

The experiment was conducted using a completely randomized design with four dietary treatments and four animals per treatment. Each animal was considered an experimental unit in this study.

Performance variables (FBW, TWG, DMI, and feed conversion ratio (FCR)) were analyzed using analysis of covariance (ANCOVA), with initial body weight (IBW) included as a covariate according to the following model:

$$\mathrm{Yij}\;=\;\mu \;+\;\mathrm{Ti}\;+\;\beta (\mathrm{Xij}\;-\;\bar{X})+\varepsilon \mathrm{ij}$$

where Yij is the observed response variable; μ is the overall mean; Ti is the fixed effect of dietary treatment; β is the regression coefficient associated with the covariate; Xij is the initial body weight of animal j; $\bar{X}$ is the overall mean initial body weight; and εij is the residual error.

In vitro fermentation variables were analyzed separately, considering substrate as the fixed effect and the fermentation flask as the experimental unit, with four flasks evaluated per substrate, according to the following model:

Yij = μ + Si + εij

where Yij is the observed response variable; μ is the overall mean; Si is the fixed effect of substrate; and εij is the residual error.

Carcass traits, carcass morphometry, commercial cuts, non-carcass components, and muscle pH were analyzed without the inclusion of a covariate, according to the following model:

Yij = μ + Ti + εij

where Yij is the observed response variable; μ is the overall mean; Ti is the fixed effect of dietary treatment; and εij is the residual error.

All analyses were performed using the PROC GLM procedure in SAS version 9.4 (SAS Institute Inc., Cary, NC, USA) [37]. Because jackfruit silage inclusion represented ordered quantitative levels (0, 333, 666, and 1000 g/kg replacement of corn), treatment responses were evaluated using orthogonal polynomial contrasts to test linear and quadratic effects of increasing jackfruit silage inclusion. The coefficients used for the linear contrast were −3, −1, 1, and 3, whereas the coefficients used for the quadratic contrast were 1, −1, −1, and 1.

Orthogonal polynomial contrasts were used as the primary inferential approach to test linear and quadratic responses to increasing jackfruit silage replacement. Regression equations fitted to treatment means were used only to describe significant response patterns. Given the four inclusion levels evaluated, regression coefficients and R^2^ values were interpreted descriptively and not as independent evidence of treatment effects.

When significant linear or quadratic effects were detected, regression equations were fitted to describe the biological response pattern. The linear model tested was:

Ŷ = β_0_ + β_1_X

and the quadratic model tested was:

Ŷ = β_0_ + β_1_X + β_2_X^2^

where Ŷ is the predicted response, X is the level of corn replacement by jackfruit silage (g/kg corn DM), β_0_ is the intercept, β_1_ is the linear regression coefficient, and β_2_ is the quadratic regression coefficient. When both models adequately described the data, preference was given to the simplest model unless inclusion of the quadratic term significantly improved model fit. The coefficient of determination (R^2^) was used as an indicator of goodness of fit and is presented for significant regressions. Statistical significance was declared at *p* ≤ 0.05, whereas 0.05 < *p* ≤ 0.10 was considered a tendency.

## 3. Results

### 3.1. In Vitro Fermentation Kinetics

No differences were detected between the substrates for FC gas volume or FC degradation rate (*p* ≥ 0.140; Table 2).

However, jackfruit silage reduced the lag time by 29.7% compared with corn (2.22 vs. 3.16 h; *p* = 0.033). Gas production from NFC represented 72.7% of that observed for corn (132.9 vs. 182.9 mL; *p* = 0.013), whereas the NFC degradation rate was 15.6% lower (0.081 vs. 0.096 h^−1^; *p* = 0.034). Consequently, total carbohydrates gas production from jackfruit silage reached approximately 80% of that obtained with corn (238.5 vs. 298.2 mL; *p* = 0.044).

**Figure 1:**
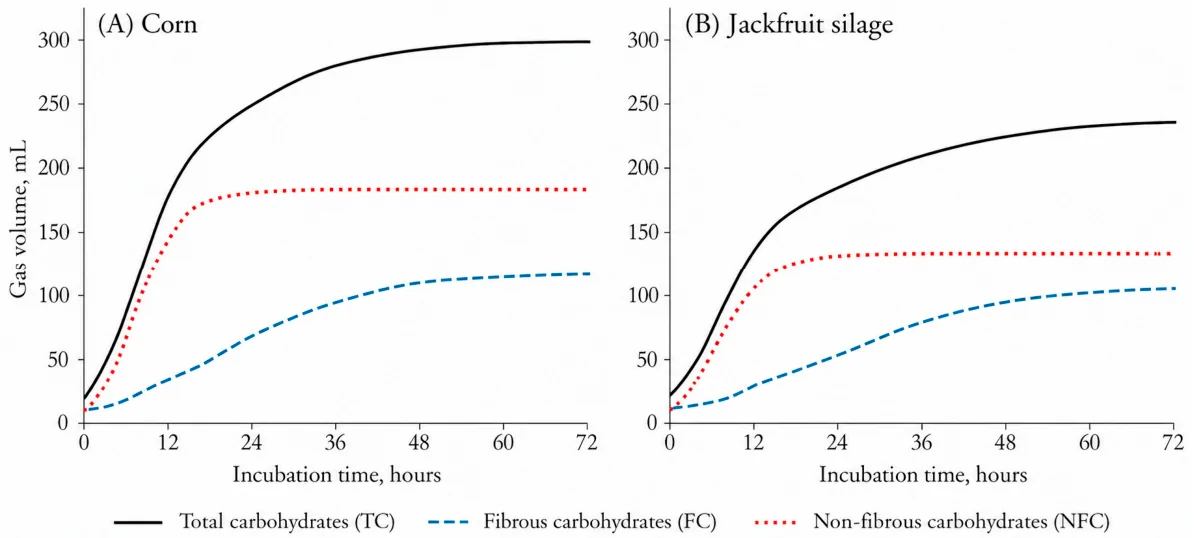
Cumulative gas production profiles of total carbohydrates (TC), fibrous carbohydrates (FC), and non-fibrous carbohydrates (NFC) obtained from in vitro fermentation of corn (**A**) and jackfruit silage (**B**) during the 72 h incubation period.

As illustrated in Figure 1, gas production increased rapidly during the first 24 h of incubation and showed only minor increases thereafter. The greatest divergence between substrates was observed for the non-fibrous carbohydrate fraction, in which corn consistently exhibited higher cumulative gas production than jackfruit silage throughout the incubation period. In contrast, the FC curve for corn showed limited additional gas accumulation after approximately 48 h, whereas the FC curve for jackfruit silage continued to increase until the end of the 72 h incubation period. In contrast, the main separation in NFC gas production occurred earlier, with corn maintaining greater cumulative gas production than jackfruit silage throughout incubation.

### 3.2. Nutrient Intake, Apparent Organic Digestibility and Performance

Final body weight decreased linearly with increasing dietary inclusion of jackfruit silage (*p* = 0.011), ranging from 38.9 kg in lambs fed the corn-based diet to 35.1 kg in those receiving complete replacement of corn (Table 3). According to the regression equation, final body weight decreased by approximately 0.394 kg for each 100 g/kg increase in corn replacement by jackfruit silage. Similarly, total weight gain decreased linearly (*p* = 0.011), declining from 12.6 to 8.8 kg, which corresponds to a reduction of approximately 0.367 kg for each 100 g/kg increase in corn replacement by jackfruit silage.

Dry matter intake increased linearly with increasing levels of jackfruit silage in the diet (*p* < 0.001), reaching 1488.0 g/day at the highest replacement level. This value represented a 22.7% increase compared to that of lambs fed the corn-based diet. Based on the regression equation, dry matter intake increased by approximately 25.7 g/day for each 100 g/kg increase in corn replacement by jackfruit silage.

The apparent organic matter digestibility decreased linearly as jackfruit silage replaced corn in the concentrate (*p* = 0.002). However, the magnitude of this response was relatively small, with digestibility declining from 634.6 to 623.3 g/kg, representing a reduction of only 1.8% between the lowest and highest replacement levels. The regression equation indicated a decrease of approximately 1.13 g/kg in digestibility for each 100 g/kg increase in corn replacement by jackfruit silage.

Feed conversion ratio increased linearly with increasing replacement of corn by jackfruit silage (*p* < 0.001), ranging from 6.6 to 12.3 kg DMI/kg gain. According to the regression equation, feed conversion worsened by approximately 0.47 units for every 100 g/kg increase in corn replacement by jackfruit silage.

Despite the negative linear responses observed at higher inclusion levels, lambs receiving 333 g/kg corn replacement by jackfruit silage maintained 98.5% of the final body weight (38.3 vs. 38.9 kg) and 96.0% of the total weight gain (12.1 vs. 12.6 kg) achieved by animals fed the conventional corn-based diet.

### 3.3. Carcass Traits

No effects of jackfruit silage inclusion were observed on slaughter body weight, empty body weight, hot carcass weight, cold carcass weight, hot carcass yield, cold carcass yield, or Longissimus muscle area (*p* ≥ 0.219; Table 4). Carcass yield values remained relatively constant across treatments, indicating that replacement of corn with jackfruit silage did not affect carcass dressing percentage.

Chilling loss decreased linearly with increasing levels of jackfruit silage in the diet (*p* = 0.022), declining from 2.6% in lambs fed the corn-based diet to 1.3% in those receiving complete replacement of corn. According to the regression equation, chilling loss decreased by approximately 0.14 percentage units for each 100 g/kg increase in corn replacement by jackfruit silage.

Subcutaneous fat thickness increased linearly with increasing jackfruit silage replacement (*p* = 0.005), from 0.68 mm in the control treatment to 1.50 mm at complete corn replacement. The fitted regression corresponded to an increase of approximately 0.086 mm in subcutaneous fat thickness for each 100 g/kg increment in jackfruit silage replacement.

Lambs receiving 333 g/kg corn replacement by jackfruit silage maintained carcass yield values comparable to those observed in animals fed the conventional corn-based diet while presenting similar Longissimus muscle area and subcutaneous fat thickness.

### 3.4. Carcass Morphometry and Muscle pH

Most carcass morphometric traits, including external carcass length, chest depth, rump width, rump perimeter, and half-carcass weight, were not affected by dietary jackfruit silage inclusion (*p* ≥ 0.307; Table 5). Internal carcass length showed a significant linear response to increasing corn replacement by jackfruit silage (*p* = 0.025), decreasing from 66.2 to 62.1 cm. According to the fitted regression equation, internal carcass length decreased by approximately 0.42 cm for each 100 g/kg increase in corn replacement by jackfruit silage.

Leg length decreased linearly as jackfruit silage replaced corn in the diet (*p* < 0.001), with treatment means of 53.1, 49.0, 47.4, and 45.8 cm for 0, 333, 666, and 1000 g/kg replacement, respectively. The fitted regression corresponded to a decrease of approximately 0.71 cm in leg length for each 100 g/kg increase in corn replacement by jackfruit silage. In contrast, leg width increased linearly (*p* = 0.011), ranging from 6.8 to 8.5 cm, corresponding to an increase of approximately 0.17 cm for each 100 g/kg increase in corn replacement by jackfruit silage.

Leg depth exhibited both linear and quadratic responses (*p* < 0.001), with the greatest values observed at intermediate inclusion levels and a marked reduction at the highest level of jackfruit silage inclusion.

Muscle pH at 0 and 24 h post-mortem showed significant linear and quadratic responses to increasing corn replacement by jackfruit silage (*p* ≤ 0.045). Initial pH ranged from 6.2 to 6.9, with the control group showing the lowest value (6.2); the cause of this pattern could not be determined from the variables measured. After 24 h of chilling, pH ranged from 5.4 to 6.1.

### 3.5. Weights and Yields of Commercial Carcass Cuts

Among the absolute weights of the commercial cuts, only the rib weight was affected by dietary treatment, decreasing linearly with increasing jackfruit silage inclusion (*p* < 0.001; Table 6). Rib weight declined from 1.9 kg in lambs fed the corn-based diet to 1.3 kg in those receiving complete replacement of corn. According to the regression equation, rib weight decreased by approximately 0.056 kg for every 100 g/kg increase in corn replacement by jackfruit silage. Lambs fed 333 g/kg corn replacement by jackfruit silage maintained approximately 89.5% of the rib weight observed in animals fed the control diet.

The weights of the neck, shoulder, rib/brisket, hind shank, loin, and half-carcass were not affected by the dietary treatment (*p* ≥ 0.281). Leg weight tended to decrease (*p* = 0.052), whereas foreshank weight (*p* = 0.067) and hind shank weight (*p* = 0.065) showed tendencies toward treatment effects.

For the relative yields of commercial cuts, rib yield decreased linearly with increasing dietary inclusion of jackfruit silage (*p* = 0.011), whereas hind shank yield increased linearly (*p* = 0.021). Shoulder yield (*p* = 0.009) and foreshank yield (*p* = 0.040) exhibited quadratic responses. No effects were observed on neck, rib/brisket, or loin yields (*p* ≥ 0.424), whereas leg yield showed a tendency toward treatment effects (*p* = 0.080).

Overall, the distribution of commercial cuts within the carcass remained largely unchanged despite the progressive replacement of corn with jackfruit silage.

### 3.6. Non-Carcass Components

Dietary replacement of corn with jackfruit silage did not affect the weights of the non-carcass components (*p* ≥ 0.102; Table 7). No significant linear or quadratic responses were detected for blood, skin, head, hooves, tail, heart, kidneys, spleen, lungs, tongue, visceral fat, or empty gastrointestinal tract weight.

Likewise, empty body weight remained unchanged across treatments, averaging 31.1 kg, indicating that increasing dietary inclusion of jackfruit silage did not alter the relative development of visceral organs or non-carcass tissues.

## 4. Discussion

### 4.1. Reduced Fermentative Energy Availability Resulting from Lower Non-Fibrous Carbohydrate Fermentation

The lower NFC gas volume (132.9 vs. 182.9 mL), NFC degradation rate (0.081 vs. 0.096 h^−1^), and total carbohydrate gas volume (238.5 vs. 298.2 mL) observed for jackfruit silage indicate a lower availability of rapidly fermentable carbohydrates than corn. Because NFC fractions constitute the primary substrate for rapid microbial fermentation in the rumen, a reduction in their fermentability directly limits the rate and extent of energy release from dietary carbohydrates. Consistent with this interpretation, Li et al. [38] reported that increasing the dietary NDF:NFC ratio from 0.48 to 1.12 reduced total gas production from 205.6 to 179.8 mL, dry matter degradation from 86.7 to 73.2%, and total VFA concentration from 111.4 to 101.7 mmol/L. Likewise, Cabral et al. [39] observed greater NFC degradation rates and gas production in highly fermentable substrates such as corn (228.9 mL gas/g DM) and cassava residue (273.2 mL gas/g DM) than in more fibrous feed resources.

The chemical composition of the jackfruit silage used in the present study, particularly its 422 g/kg DM NDF and 394 g/kg DM NFC concentrations, is consistent with the progressive increase in dietary fiber and reduction in dietary NFC as corn was replaced. This compositional shift provides additional context for the lower NFC gas volume and degradation rate observed for jackfruit silage and for the subsequent reductions in productive efficiency at higher replacement levels.

The magnitude of the differences observed in the present study reinforces this hypothesis. Jackfruit silage generated only 72.7% of the NFC gas volume and approximately 80.0% of the total carbohydrate gas volume obtained with corn, while its NFC degradation rate was 15.6% lower. These results indicate that the carbohydrates present in jackfruit silage were fermented more slowly and less extensively, reducing the immediate availability of fermentable energy to ruminal microorganisms.

From a physiological perspective, this response may be relevant because ruminal fermentation is a major pathway through which ruminants obtain energy from dietary carbohydrates. Evidence from Li et al. [38] indicates that reductions in NFC availability may be accompanied by lower total VFA and propionate concentrations and reduced microbial crude protein production. However, VFA concentration and profile, ruminal pH, and microbial protein synthesis were not measured in the present study. Therefore, these mechanisms should be interpreted as literature-supported explanations for the observed responses rather than as direct findings of this experiment.

The shorter lag time observed for jackfruit silage should not be interpreted as evidence of greater overall fermentability. In the present study, jackfruit silage had a shorter lag time than corn (2.22 vs. 3.16 h) but produced less NFC gas (132.9 vs. 182.9 mL) and lower total carbohydrate gas volume (238.5 vs. 298.2 mL). This indicates that microbial colonization began earlier, but the subsequent extent of carbohydrate fermentation was lower. This interpretation is supported by Poveda-Parra et al. [40], who observed that increasing Crambe cake inclusion reduced lag time from 4.17 to 3.19 h while simultaneously decreasing final gas production from 365.0 to 233.6 mL. Similarly, Cabral et al. [39] reported that Cupuaçu meal had the shortest lag time (3.40 h) but the lowest final gas volume (63.8 mL/g DM), whereas cassava residue showed a longer lag time (15.24 h) but the highest final gas volume (273.2 mL/g DM). Therefore, lag time should be interpreted as an indicator of the onset of microbial colonization, whereas gas volume and degradation rate better represent the extent and intensity of substrate fermentation after colonization.

Collectively, the lower NFC gas volume, NFC degradation rate, and total carbohydrate gas volume indicate that jackfruit silage had lower gas-producing fermentative potential than corn under the conditions of the in vitro assay. Evidence from the cited literature suggests that lower availability of rapidly fermentable carbohydrates may be associated with reductions in VFA production, microbial protein synthesis, and energy availability [38]. Because these variables were not measured in the present study, they are considered possible explanatory mechanisms rather than experimentally demonstrated responses.

Importantly, the in vitro responses observed in this study are consistent with evidence linking gas-production kinetics to nutrient utilization and animal performance. Blümmel and Ørskov [41] reported associations between gas-production variables, dry matter intake, digestible dry matter intake, and growth rate, whereas Li et al. [42] reported associations between gas production and nutrient digestibility and fermentation variables. Accordingly, the lower NFC gas volume, NFC degradation rate, and total carbohydrate gas volume observed for jackfruit silage may help explain, but do not independently demonstrate, the reductions in organic matter digestibility and growth performance observed in vivo. The in vitro assay should therefore be interpreted as complementary evidence of differences in fermentative potential between the two ingredients.

### 4.2. Increased Feed Intake Partially Compensated for the Lower Dietary Energy Availability, but Could Not Prevent Reductions in Growth Performance

The linear increase in dry matter intake (DMI) observed with increasing dietary inclusion of jackfruit silage may represent a compensatory intake response to the lower NFC gas production and degradation rate observed for jackfruit silage in vitro.

As corn was progressively replaced, DMI increased from 1212.8 to 1488.0 g/day, representing an increase of approximately 22.7%. Similar responses have been reported in studies evaluating jackfruit-derived feeds and other alternative tropical feed resources, in which animals increased feed consumption when diets contained lower concentrations of readily fermentable carbohydrates. Das and Ghosh [20] reported that replacing 25 and 50% of concentrate with jackfruit leaves increased total DMI from 408.4 to 431.3 and 426.6 g/day, respectively, and increased DMI as a percentage of body weight from 4.73 to 5.07 and 5.73%. Similarly, Thanh et al. [21] observed a linear increase in total DMI when jackfruit leaves replaced Para grass in lactating goat diets. These comparisons support a compensatory interpretation, although the mechanisms regulating intake were not directly evaluated in the present study.

Dietary DM concentration decreased as jackfruit silage inclusion increased, resulting in progressively wetter diets. Therefore, dietary moisture changed concurrently with ingredient replacement and cannot be completely separated from the treatment effect. Nevertheless, DMI increased rather than decreased across treatments, indicating that the greater dietary moisture did not restrict voluntary DM intake under the conditions evaluated.

The increased DMI observed in the present study was consistent with the lower NFC gas volume, NFC degradation rate, and total carbohydrate gas volume observed for jackfruit silage in vitro. Wang et al. [43] reported that differences in dietary energy supply may influence VFA production, propionate concentration, nutrient digestibility, growth, and feed conversion. However, VFA concentration and profile were not measured in the present study. Thus, the increase in DMI may be interpreted as a possible compensatory response to the lower fermentative potential of jackfruit silage, rather than as evidence of a specific change in ruminal VFA production.

Organic matter digestibility decreased linearly as jackfruit silage replaced corn in the concentrate feed. However, the biological magnitude of this reduction was small, declining from 634.6 to 623.3 g/kg, corresponding to a reduction of only 11.3 g/kg or approximately 1.8% across the entire range of dietary inclusion. This distinction between statistical significance and biological relevance is important because the magnitude of the digestibility response alone is unlikely to fully explain the reductions observed in animal performance. A similar pattern of increased intake associated with reduced digestibility was reported by [21], who observed that replacing Para grass with jackfruit leaves increased total DMI from 1489 to 2140 g/day, but reduced DM digestibility from 66.4 to 58.8%, OM digestibility from 68.6 to 62.8%, CP digestibility from 78.2 to 58.8%, and NDF digestibility from 59.2 to 38.6%. Therefore, the reduction in organic matter digestibility observed in the present study indicates a modest decline in overall organic matter utilization but does not identify the specific nutrients or ruminal mechanisms responsible for the response.

The reductions in final body weight and total weight gain, together with the marked increase in feed conversion ratio, indicate that the additional feed consumed was insufficient to offset the lower productive efficiency of the diets. Feed conversion ratio increased from 6.6 to 12.3 kg DMI/kg gain as jackfruit silage replaced corn, representing an increase of approximately 86%. Thus, although lambs consumed more feed, the efficiency with which consumed DM was converted into body weight gain declined. Wang et al. [43] reported associations among dietary energy concentration, digestibility, VFA production, propionate concentration, growth, and feed conversion. In the present study, however, only intake, organic matter digestibility, growth performance, and in vitro gas-production kinetics were measured. Therefore, changes in VFA production, propionate concentration, and microbial protein synthesis remain possible mechanisms supported by the literature, but were not directly demonstrated.

Importantly, the practical interpretation of these results should focus on the partial rather than complete replacement of corn. At 333 g/kg replacement, growth performance remained close to that obtained with the conventional corn-based diet, whereas higher inclusion levels progressively impaired performance. This indicates that the nutritional value of jackfruit silage depends on identifying a biologically appropriate inclusion level rather than maximizing corn replacement. Similar threshold responses have been reported in studies evaluating jackfruit-derived feeds and other alternative feed resources for livestock. Das and Ghosh [20] observed that 25% replacement of concentrate with jackfruit leaves maintained ADG in grazing kids (47.33 vs. 45.11 g/day), whereas 50% replacement reduced ADG to 33.56 g/day. Similarly, Thi Mui et al. [19] reported that replacing up to 50% of concentrate protein with jackfruit foliage maintained growth rate in growing goats (57, 53, and 58 g/day at 0, 25, and 50% replacement), but higher replacement levels reduced growth to 44 and 30 g/day at 75 and 100%, respectively.

These findings indicate that the nutritional value of jackfruit silage should not be judged based on its ability to completely replace corn. Rather, its practical importance lies in its capacity to partially substitute a conventional energy source while preserving most of the productive response. The increase in feed intake partially compensated for the lower fermentative energy availability of the diets, allowing animals receiving moderate levels of jackfruit silage to maintain growth performance close to that obtained with the conventional diet. Thus, moderate inclusion of jackfruit silage partially compensated for its lower fermentative potential while preserving productive viability, whereas complete replacement was not supported by the performance responses.

### 4.3. Preservation of Carcass Value Despite Reduced Growth Performance

Although increasing levels of jackfruit silage reduced final body weight and total weight gain, the principal carcass characteristics associated with commercial value were largely preserved. Hot carcass yield, cold carcass yield, hot carcass weight, cold carcass weight, and Longissimus muscle area were not affected by dietary treatment. These results indicate that the reduction in animal growth did not translate into proportional reductions in carcass yield or muscle mass. A similar response was reported by [44], who observed that increasing dietary metabolizable energy from 9.17 to 10.41 MJ/kg increased live weight before slaughter from 25.36 to 27.44 kg and carcass weight from 10.74 to 11.82 kg, but did not affect slaughter ratio (42.38 vs. 43.08%). This supports the interpretation that dietary energy supply may alter total tissue accretion more clearly than carcass yield, which reflects the proportional relationship between carcass weight and slaughter body weight.

The maintenance of carcass yield despite reductions in body weight gain suggests that carcass and non-carcass tissues were affected proportionally by the progressive replacement of corn. Consequently, animals receiving higher levels of jackfruit silage produced lighter carcasses because they were lighter at slaughter; however, the proportion of carcass relative to slaughter weight remained unchanged. This interpretation is consistent with evidence indicating that carcass dressing percentage is largely determined by body composition and gastrointestinal fill and may remain relatively stable despite moderate differences in nutrient intake and growth rate [44].

An interesting response observed in the present study was the simultaneous increase in subcutaneous fat thickness and reduction in chilling loss as jackfruit silage inclusion increased. Subcutaneous fat thickness increased linearly from 0.68 to 1.50 mm, whereas the chilling loss decreased from 2.6 to 1.3%. These responses appear physiologically related because subcutaneous fat acts as a thermal and physical barrier that reduces water evaporation during carcass chilling. Consequently, greater fat cover may have contributed to the lower moisture loss observed during refrigerated storage. Although carcasses from lambs fed higher levels of jackfruit silage were lighter, the increased fat cover may have partially compensated for the potential reductions in carcass protection during chilling.

The absence of treatment effects on Longissimus muscle area further indicates that muscular development was largely preserved despite the reductions in growth performance. The rib eye area is widely used as an indicator of carcass muscularity and meat yield potential and is therefore considered an important determinant of commercial carcass quality. Similarly, although both initial and ultimate muscle pH exhibited significant responses to dietary treatment, all values remained within the physiological range commonly reported for lamb carcasses. The absence of abnormal pH values suggests that post-mortem glycolysis occurred normally and provides no evidence that dietary jackfruit silage adversely affected muscle metabolism or carcass quality of the lambs.

Among the commercial cuts, rib weight was the only cut that clearly decreased with increasing dietary inclusion of jackfruit silage, declining from 1.9 to 1.3 kg. Rib yield also decreased linearly, whereas shoulder, foreshank, and hind shank yields exhibited statistical responses. However, these changes were relatively small when compared with the overall stability observed for carcass yield, muscularity, and the majority of commercial cuts. Therefore, despite some alterations in the relative contribution of specific anatomical regions, the overall distribution of carcass tissues remained largely unchanged. Similar responses were reported by [45], who observed that replacing sorghum silage with corn straw reduced ADG from 0.23 to 0.20 kg/day, rib weight from 2.92 to 2.37 kg, and rib yield from 28.34 to 25.93%, while shoulder, neck, loin, and leg weights, loin eye area, subcutaneous fat thickness, leg muscle index, leg tissue composition, and meat physicochemical traits were not affected. This supports the interpretation that alternative feed resources may reduce growth and specific cut weights without substantially altering the overall distribution of carcass tissues.

Likewise, the absence of treatment effects on non-carcass components indicates that visceral development was maintained despite the replacement of corn by jackfruit silage. In the present study, the weights of the heart, kidneys, spleen, lungs, empty gastrointestinal tract, tongue, and visceral fat were not affected, suggesting that the dietary treatments did not produce detectable alterations in organ development. This interpretation is relevant because metabolically active organs may respond to changes in nutrient supply and feeding systems. Morais et al. [46], for example, observed that replacing ground corn with a blend of candy industry residue and corn gluten feed increased heart weight from 154.15 to 172.18 g, liver weight from 576.45 to 650.18 g, and rumen weight from 670.36 to 738.69 g. Similarly, Silva et al. [47] reported that feedlot lambs had greater liver, heart, spleen, kidney plus perirenal fat, and omental fat yields than lambs finished on pasture, increasing to 1.77, 0.44, 0.18, 1.21, and 1.24% of slaughter body weight, respectively, whereas empty gastrointestinal tract and lungs plus trachea were not affected. Therefore, the lack of changes in non-carcass components in the present study supports the interpretation that jackfruit silage replacement did not impose detectable physiological changes in visceral development under the conditions evaluated.

From a practical perspective, these findings are highly relevant because economic returns in lamb production depend not only on growth rate but also on carcass quality and commercial value. Although complete replacement of corn reduced animal performance, the principal carcass characteristics remained largely unchanged. Despite the reduction in growth performance at higher replacement levels, the principal carcass traits associated with commercial value were largely preserved.

### 4.4. Partial Replacement of Corn with Jackfruit Silage Improves Resource-Use Efficiency and May Support Feed-System Resilience

The practical relevance of jackfruit silage extends beyond its nutritional value and should be interpreted within the broader context of resource-use efficiency and feed-system resilience. At the biologically viable replacement level, corn use per kilogram of body weight gain was reduced by 26.2% while productive and carcass responses remained close to those obtained with the conventional diet. This indicates that partial replacement can reduce reliance on conventional feed grain without requiring complete substitution.

The importance of this finding lies not in the complete replacement of corn, but in the identification of a biologically viable replacement level. This interpretation is consistent with studies showing that alternative feed resources can partially replace conventional energy feeds when productive responses are preserved. Dentinho et al. [48] reported that replacing 50% of the dry matter of a concentrate-based diet with agro-industrial by-product silages did not affect DMI (1024–1099 g/day), ADG (303–334 g/day), feed conversion ratio (3.14–3.56 kg DM/kg gain), hot carcass weight (16.5–17.3 kg), cold carcass weight (16.0–16.8 kg), or dressing percentage (48.5–49.6%), while reducing feed cost per kilogram of live weight gain from €1.40 to €1.03–1.16. Likewise, Arruda et al. [49] observed that soybean molasses replacing corn up to 30% of diet DM maintained final body weight (34.4–35.9 kg), total ADG (0.23–0.28 kg/day), hot carcass weight (15.6–16.8 kg), cold carcass weight (15.3–16.2 kg), carcass yield (41.5–45.4%), and Longissimus muscle area (11.0–12.1 cm^2^). In goats, Rahman et al. [50] also reported that replacing corn with dried citrus pulp up to 20% of the total mixed ration did not affect feed intake, weight gain, or feed conversion ratio, while reducing diet cost from 32.37 to 29.80 PKR/kg. Collectively, these findings support the concept that the value of alternative feed resources depends on identifying inclusion levels that maintain productive viability rather than pursuing complete replacement of conventional grains.

The present findings also contribute to the growing interest in valorizing locally available biomass resources for feeding small ruminants. Anim-Jnr et al. [51] identified mango, cashew apple, and papaya by-products as promising feed resources for small ruminants, including mango peels and seeds, cashew apple pomace, and papaya peels and seeds. The scale of biomass generation is substantial: mango processing may generate 35–55% of fruit mass as by-products, cashew nut production may produce 36.9 million tons of cashew apple waste annually, and papaya processing may generate by-products equivalent to up to 20% of fruit weight. These data support the concept that fruit-derived biomass can be redirected from low-value waste streams to animal feeding systems. Similarly, Dentinho et al. [48] showed that agro-industrial by-product silages prepared from tomato pomace combined with potato, sweet potato, or carrot wastes could replace 50% of the dry matter of a concentrate-based lamb diet without impairing DMI, ADG, feed conversion, carcass traits, or meat quality, while reducing feed cost per kilogram of live weight gain from €1.40 to €1.03–1.16. In this context, jackfruit silage represents a practical example of biomass valorization, transforming an underutilized tropical resource into a feed ingredient capable of reducing dependence on conventional grain while maintaining productive viability at biologically appropriate inclusion levels.

An important distinction should be made between sustainability and resilience. Sustainability refers to the ability to maintain production while reducing dependence on conventional resources and improving resource-use efficiencies. Resilience, in contrast, reflects the capacity of livestock systems to continue functioning under conditions of feed scarcity, price volatility, supply-chain disruptions, or limited access to conventional feed ingredients. The results obtained in the present study indicate that partial replacement of corn with jackfruit silage may contribute to feed-system resilience by reducing dependence on a single conventional feed commodity while maintaining productive viability. This interpretation is consistent with recent reviews emphasizing feed diversification and the incorporation of locally available biomass resources as important strategies for reducing the vulnerability of livestock systems to fluctuations in feed availability and cost [4,51].

Although environmental indicators were not directly measured in the present study, the reduction in corn requirements per unit of productive output clearly demonstrates improved resource-use efficiency. Thus, the benefits identified here should be interpreted in terms of reduced dependence on conventional feed resources rather than direct environmental mitigation effects. This distinction is important because it ensures that the conclusions are fully supported by the experimental data.

Although complete replacement of corn reduced growth performance, partial replacement proved to be a viable strategy. Lambs receiving 333 g/kg corn replacement by jackfruit silage maintained nearly the same final body weight, total weight gain, carcass yields, and muscularity as animals fed the conventional corn-based diet while requiring substantially less corn per unit of productive output. Therefore, the value of jackfruit silage should not be assessed according to its ability to completely replace corn, but rather according to its capacity to partially substitute a conventional feed grain while preserving productive performance and carcass value.

Collectively, these findings support jackfruit silage as a strategic partial substitute for corn in tropical feeding systems, particularly where locally available biomass can reduce reliance on conventional feed commodities.

### 4.5. Study Limitations and Practical Implications

The present study evaluated the effects of replacing corn with jackfruit silage on ruminal fermentation kinetics, nutrient utilization, growth performance, carcass characteristics, and non-carcass components of feedlot lambs under controlled experimental conditions. Therefore, the conclusions should be interpreted within the biological and production conditions evaluated in this study. The experiment was conducted using growing lambs fed a single forage source and a fixed forage-to-concentrate ratio, and the responses observed may differ under alternative feeding systems, production objectives, forage sources, or animal categories.

In addition, the present study focused primarily on productive and carcass responses and was not designed to evaluate long-term animal health, ruminal microbial populations, enteric methane emissions, life-cycle environmental impacts, or the economic consequences of replacing corn with jackfruit silage. Consequently, although the results support improvements in resource-use efficiency through reduced corn utilization, direct inferences regarding greenhouse gas mitigation, environmental footprints, or economic returns cannot be made from the present dataset. These aspects should be addressed in future studies to provide a more comprehensive assessment of the practical implications of incorporating jackfruit silage into livestock production systems.

Despite these limitations, the directionally coherent responses across in vitro fermentation, organic matter digestibility, performance, and carcass measurements support a consistent interpretation of the dataset. However, the in vitro findings remain ingredient-specific and should not be considered independent biological validation of the in vivo responses.

The in vitro fermentation assay was performed using the two principal energy ingredients (corn and jackfruit silage) rather than the complete experimental diets. Consequently, the fermentation parameters should be interpreted as ingredient-specific responses intended to characterize the fermentative potential of the energy sources and not as direct predictions of the fermentation dynamics of the complete diets.

Ruminal fluid from two donor sheep was pooled and used uniformly across all fermentation flasks. Therefore, the in vitro comparison represents the relative fermentation responses of corn and jackfruit silage under a common standardized inoculum. The assay was conducted within a single incubation run; consequently, variation among independent donor inocula and incubation runs was not evaluated. Because the donor sheep were fed the corn-based control diet, the inoculum was adapted to that feeding condition. Therefore, the in vitro results should be interpreted as a relative comparison of the fermentative potential of corn and jackfruit silage under a common standardized inoculum, rather than as a direct representation of ruminal fermentation in animals adapted to diets containing jackfruit silage.

Another limitation of the present study is that detailed fermentative quality parameters of jackfruit silage, including pH, organic acid concentrations, ammonia nitrogen, and fermentative losses, were not determined. No formal organoleptic assessment of smell, color, or texture, or microbiological assessment of fungal contamination, was performed. Therefore, a direct assessment of silage fermentation quality and preservation losses was not possible. Nevertheless, the absence of reductions in voluntary feed intake suggests that the silage did not exhibit sensory characteristics severe enough to impair acceptance by the animals under the conditions evaluated. Future studies should incorporate comprehensive fermentative quality assessments to better characterize the relationship between silage fermentation profile, nutrient utilization, and animal performance.

## 5. Conclusions

Partial replacement of corn with jackfruit silage was more appropriate than complete replacement under the conditions evaluated. Replacing 333 g/kg of corn maintained 98.5% of final body weight and 96.0% of total weight gain, while carcass yield and Longissimus muscle area were preserved and corn use per kilogram of gain was reduced by 26.2%. Complete replacement reduced productive performance and increased feed conversion ratio. Therefore, jackfruit silage is better suited as a strategic partial substitute for corn in feedlot lamb diets.

## Figures and Tables

**Table 1:** Ingredient composition and chemical composition of experimental diets.

Item	Corn Replacement by Jackfruit Silage (g/kg Corn DM)			
	0	333	666	1000
Ingredient composition (g/kg DM)				
Elephant grass silage	400	400	400	400
Ground corn	370	247	123	0
Jackfruit silage	0	123	247	370
Soybean meal	205	205	205	205
Urea/ammonium sulfate (9:1)	10	10	10	10
Commercial premix ^1^	15	15	15	15
Chemical composition (g/kg DM)				
Dry matter, g/kg fresh matter	649.3	575.6	501.3	427.6
Organic matter	930.1	924.6	919.0	913.4
Crude protein	157.8	156.9	156.1	155.2
Ether extract	28.4	25.4	22.3	19.3
NDFap ^2^	339.3	354.5	369.8	385.1
Non-fibrous carbohydrates	407.4	390.5	373.4	356.5
Total digestible nutrients ^3^	755.3	744.5	733.6	722.8

^1^ Guaranteed composition (per kg): calcium, 82 g; phosphorus, 60 g; sulfur, 11.7 g; magnesium, 13 g; sodium, 132 g; copper, 350 mg; cobalt, 30 mg; chromium, 11.7 mg; iron, 700 mg; iodine, 50 mg; manganese, 1200 mg; selenium, 15 mg; zinc, 2600 mg; maximum fluorine, 600 mg. ^2^ NDFap: Neutral detergent fiber corrected for ash and protein; ^3^ Nutrient composition and Total digestible nutrients values were estimated according to [28].

**Table 2:** In vitro gas production kinetics parameters of corn and jackfruit silage.

Parameters	Jackfruit Silage	Corn	Means	SEM	*p*-Value
FC gas volume, mL	105.6	115.3	110.4	8.1	0.655
FC degradation rate, h^−1^	0.026	0.030	0.028	0.001	0.140
Lag time, h	2.22	3.16	2.7	0.3	0.033
NFC gas volume, mL	132.9	182.9	157.9	14.6	0.013
NFC degradation rate, h^−1^	0.081	0.096	0.089	0.005	0.034
TC gas volume, mL	238.5	298.2	268.3	18.0	0.044

FC: fibrous carbohydrates; NFC: non-fibrous carbohydrates; TC gas volume: Total carbohydrates gas volume calculated as the sum of gas produced from fibrous and non-fibrous carbohydrate fractions. SEM: standard error of the mean.

**Table 3:** Intake, apparent organic digestibility, and performance of feedlot lambs fed diets with increasing levels of jackfruit silage replacing corn.

Item	Corn Replacement by Jackfruit Silage (g/kg Corn DM)				SEM	*p*-Value		Regression Equation
	0	333	666	1000		L	Q	
Initial BW, kg	26.1	26.5	25.5	26.6	0.8	---	---	-
Final BW, kg	38.9	38.3	35.9	35.1	1.1	0.011	0.424	Ŷ = 39.0156 − 0.00394X
Total WG, kg	12.6	12.1	9.6	8.8	0.8	0.011	0.424	Ŷ = 12.604 − 0.00367X
DMI, g/day	1212.8	1231.6	1428.8	1488.0	23.2	<0.001	0.693	Ŷ = 1197.71 + 0.25681X
OM digestibility, g/kg	634.6	636.8	633.2	623.3	1.4	0.002	0.602	Ŷ = 637.5746 − 0.0113X
FCR, kg DMI/kg gain	6.6	7.3	9.6	12.3	0.7	<0.001	0.352	Ŷ = 7.2574 + 0.00466X

R^2^ values corresponding to Equations (from top to bottom) were 0.94, 0.93, 0.91, 0.66, and 0.95, respectively. SEM = standard error of the mean; FCR = feed conversion ratio; X = corn replacement by jackfruit silage (g/kg corn DM).

**Table 4:** Slaughter and carcass traits of feedlot lambs fed diets with increasing levels of jackfruit silage replacing corn.

Item	Corn Replacement by Jackfruit Silage (g/kg Corn DM)				SEM	*p*-Value		Regression Equation
	0	333	666	1000		L	Q	
Slaughter weight, kg	36.9	36.1	34.9	33.4	1.1	0.277	0.868	---
Empty body weight, kg	32.3	31.6	31.7	29.8	1.0	0.462	0.773	---
Hot carcass weight, kg	18.2	18.4	18.1	16.7	0.6	0.459	0.589	---
Cold carcass weight, kg	17.7	18.0	17.8	16.4	0.6	0.534	0.573	---
Hot carcass yield, %	49.2	50.8	51.9	49.7	0.7	0.691	0.219	---
Cold carcass yield, %	47.9	49.8	51.1	49.0	0.7	0.472	0.209	---
Chilling loss, %	2.6	2.1	1.5	1.3	0.2	0.022	0.616	Ŷ = 2.5555 − 0.001367X
Subcutaneous fat thickness, mm	0.7	1.0	1.4	1.5	0.2	0.005	0.459	Ŷ = 0.732 + 0.00086X
Longissimus muscle area, cm^2^	14.8	16.6	15.9	14.8	0.7	0.904	0.359	

R^2^ values corresponding to Equations (from top to bottom) were 0.97 and 0.94, respectively. SEM = standard error of the mean; L = linear; Q = quadratic; X = corn replacement by jackfruit silage (g/kg corn DM).

**Table 5:** Carcass morphometric measurements and muscle pH of feedlot lambs fed diets with increasing levels of jackfruit silage replacing corn.

Item	Corn Replacement by Jackfruit Silage (g/kg Corn DM)				SEM	*p*-Value		Regression Equation
	0	333	666	1000		L	Q	
Internal carcass length, cm	66.2	64.3	62.9	62.1	1.2	0.025	0.674	Ŷ = 65.9664 − 0.00418X
External carcass length, cm	110.2	107.8	106.0	106.2	2.5	0.235	0.605	---
Leg length, cm	53.1	49.0	47.4	45.8	1.0	<0.001	0.223	Ŷ = 52.365 − 0.00709X
Chest depth, cm	29.3	28.6	29.0	27.9	0.5	0.400	0.893	---
Rump width, cm	16.2	17.2	17.5	18.1	0.6	0.360	0.916	---
Rump perimeter, cm	52.8	54.8	55.0	57.2	1.3	0.307	0.969	---
Half-carcass weight, kg	8.3	8.4	8.3	7.3	0.3	0.341	0.459	---
Leg width, cm	6.8	7.0	7.6	8.5	0.3	0.011	0.448	Ŷ = 6.66231 + 0.0017X
Leg depth, cm	10.8	11.3	10.3	7.6	0.4	<0.001	<0.001	Ŷ = 10.76488 + 0.00341X − 0.00000657X^2^
pH at 0 h post-mortem	6.2	6.9	6.7	6.7	0.1	0.045	0.031	Ŷ = 6.2792 + 0.00176X − 0.00000139X^2^
pH at 24 h post-mortem	5.9	6.1	5.8	5.4	0.1	<0.001	0.010	Ŷ = 6.03838 − 0.00050174X

R^2^ values corresponding to Equations (from top to bottom) were 0.97, 0.94, 0.93, 0.99, 0.77, and 0.62, respectively. SEM = standard error of the mean; L = linear; Q = quadratic; X = corn replacement by jackfruit silage (g/kg corn DM).

**Table 6:** Weights and yields of primal carcass cuts of feedlot lambs fed diets with increasing levels of jackfruit silage replacing corn.

Item	Corn Replacement by Jackfruit Silage (g/kg Corn DM)				SEM	*p*-Value		Regression Equation
	0	333	666	1000		L	Q	
Weight, kg								
Half-carcass	8.3	8.2	8.1	7.4	0.30	0.281	0.569	---
Neck	1.0	0.9	0.9	0.9	0.04	0.435	0.735	---
Shoulder	1.3	1.1	1.1	1.2	0.10	0.592	0.250	---
Rib	1.9	1.7	1.5	1.3	0.10	<0.001	0.628	Ŷ = 1.87853 − 0.00056485X
Leg	2.2	2.2	2.2	1.7	0.10	0.052	0.099	---
Foreshank	0.37	0.39	0.41	0.32	0.03	0.416	0.067	---
Rib/Brisket	1.7	1.7	1.8	1.6	0.10	0.562	0.533	---
Hind Shank	0.30	0.40	0.40	0.40	0.03	0.065	0.445	---
Loin	0.58	0.58	0.61	0.55	0.02	0.782	0.612	---
Yield, %								
Neck	12.4	11.5	11.2	12.4	0.6	0.954	0.424	---
Shoulder	15.4	13.7	13.1	16.1	0.5	0.703	0.009	Ŷ = 15.5692 − 0.01013X + 0.00001052X^2^
Rib	22.8	20.8	18.7	17.7	0.8	0.011	0.718	Ŷ = 22.60914 − 0.00526X
Leg	26.8	27.1	27.7	22.4	0.9	0.080	0.081	---
Foreshank	4.5	4.8	5.1	4.4	0.1	0.847	0.040	Ŷ = 4.44094 + 0.00218X − 0.00000213X^2^
Rib/Brisket	20.8	20.9	22.1	21.2	0.4	0.578	0.616	---
Hind Shank	4.1	4.5	4.4	5.9	0.3	0.021	0.205	Ŷ = 3.93025 + 0.00159X
Loin	7.0	7.0	7.5	7.5	0.3	0.492	0.940	---

R^2^ values corresponding to Equations (from top to bottom) were 0.99, 0.95, 0.98, 0.83, and 0.73, respectively. SEM = standard error of the mean; X = corn replacement by jackfruit silage (g/kg corn DM).

**Table 7:** Weights of non-carcass components of feedlot lambs fed diets with increasing levels of jackfruit silage replacing corn.

Item	Corn Replacement by Jackfruit Silage (g/kg Corn DM)				SEM	*p*-Value	
	0	333	666	1000		L	Q
Weight, kg							
Empty body weight	32.3	31.1	31.5	29.6	0.80	0.331	0.814
Blood	1.6	1.6	1.5	1.4	0.04	0.251	0.996
Skin	3.6	3.4	3.3	3.3	0.10	0.235	0.996
Head	1.4	1.5	1.5	1.5	0.04	0.638	0.897
Hooves	0.8	0.9	0.8	0.8	0.01	0.120	0.339
Tail	0.09	0.09	0.11	0.12	0.01	0.197	0.507
Heart	0.19	0.15	0.15	0.14	0.01	0.102	0.519
Kidneys	0.10	0.11	0.11	0.09	0.01	0.519	0.444
Spleen	0.07	0.06	0.07	0.07	0.01	0.268	0.806
Lungs	0.4	0.3	0.4	0.4	0.01	0.602	0.686
Empty gastrointestinal tract	3.4	3.0	3.0	2.7	0.10	0.245	0.880
Tongue	0.1	0.1	0.1	0.1	0.01	0.715	0.870
Visceral fat	1.6	1.3	1.5	1.3	0.10	0.547	0.917

SEM = standard error of the mean.

## Data Availability

The raw data supporting the conclusions of this article will be made available by the authors on request.

## Abbreviations

FCGV	Gas volume derived from the fibrous carbohydrate fraction
FCDR	Degradation rate of the fibrous carbohydrate fraction
NFCGV	Gas volume derived from the non-fibrous carbohydrate fraction
NFCDR	Degradation rate of the non-fibrous carbohydrate fraction
FC	Fibrous carbohydrates
NFC	Non-fibrous carbohydrates
CP	Crude protein
DM	Dry matter
DMI	Dry matter intake
OM	Organic matter
OMI	Organic matter intake
EE	Ether extract
MM	Mineral matter
NDFap	Neutral detergent fiber corrected for ash and protein
ADF	Acid detergent fiber
TDN	Total digestible nutrients
IBW	Initial body weight
FBW	Final body weight
TWG	Total weight gain
FCR	Feed Conversion Ratio
ADG	Average daily gain
SBW	Slaughter body weight
EBW	Empty body weight
HCW	Hot carcass weight
HCY	Hot carcass yield
CCW	Cold carcass weight
CCY	Cold carcass yield
CL	Chilling loss
GIT	Gastrointestinal tract
ANCOVA	Analysis of covariance
SEM	Standard Error of the Mean
TC	Total carbohydrates
VFA	Volatile fatty acids
